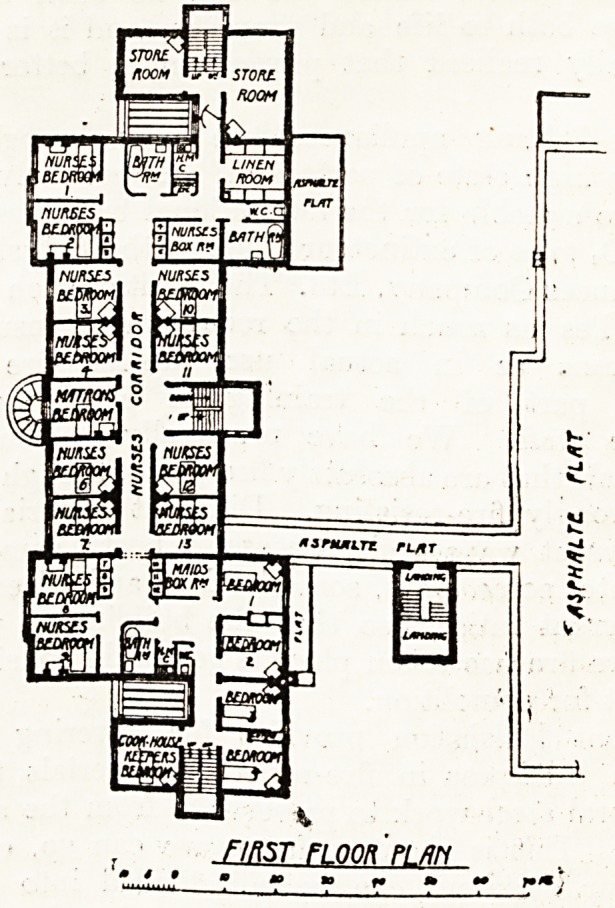# The Weir Cottage Hospital, Balham, S.W.

**Published:** 1912-11-30

**Authors:** 


### HOSPITAL ARCHITECTURE AND CONSTRUCTION.

[Communications on this subject should be marked "Architecture" in the left-hand top corner of the envelope.]

### The Weir Cottage Hospital, Balham, S.W.

The controversy which arose over the disposition of the large fund left by the late Mr. Weir to found a cottage hospital for Balham and the neighbourhood will be in the memory of our readers. The building which we illustrate to-day is the outcome of the decision of the Court of Appeal upsetting the scheme promulgated by the Charity Commissioners, which allocated a considerable portion of the fund to the completion of the Bolingbroke Hospital instead of founding another new hospital. One of the grounds upon which this decision was given was that the hospital was to be a "cottage" hospital. Whether the building which is now in hand can by any possibility be called a cottage hospital is certainly more than doubtful.

### The Building and the Site.

In the present scheme the total amount of beds is thirty, and provision is made for doubling this number. The new hospital is to be erected on the site of Nos. 15 and 17 Grove Road, Clapham Park, but we are unfortunately without any information as to the area of the site or the nature of the immediate surroundings. The building, as planned, comprises three distinct blocks connected by a corridor.

The administration block is to the south and is two storeys in height, containing on the ground floor, to the left of the central entrance, waiting room, secretary's office, and board room in front, while at the back the medical officer's sitting room, bedroom, bath, etc., are placed together with a separate entrance from the outside. Closely adjoining this is the out-patients' entrance, with waiting room and dispensary, one consulting room, and two examining rooms. On the right of the entrance is the porter's bed and sitting room, matron's room and nurses' dining room in front, with a receiving room for

GROUND FLOOR PLAN.

patients at the back. The kitchen, offices, and tradesmen's entrance are planned at the eastern

FIRST FLOOR PLAN

end of this block. The position of the porter's bedroom opening into the entrance hall and immediately adjoining the matron's sitting room does not seem at all a desirable or appropriate arrangement.

A corridor leads from the back of the administration block northwards to the two ward blocks, the first being the male block, which comprises a ward of ten beds and one of two, ward kitchen, bath room, linen room, and patients' clothes store, with the sanitary offices off the entrance end of the ward, planned very much like that at the Bolingbroke Hospital. At the western end of the ward is a wide verandah, with steps leading into the garden.

### The Theatre Unit.

On the east side of the corridor is the operation block, consisting of an anæsthetic room, operation room and preparation room, pathological and testing room, *x*-ray room, dark room, and store on the other side. It would appear that a patient after an operation must be taken back to the ward through the anæsthetic room. This is a very unfortunate arrangement, and could easily have been avoided by making the central passage wider. The female ward is arranged in the same way as the male, but in place of the operation block is a children's ward for five beds, with bath room and the sanitary offices. An open covered way at the end of the corridor leads to an observation ward, with a ward kitchen and sanitary offices. The upper floor over the administrative block consists of matron's bedroom and bedrooms for twelve nurses, cook-housekeeper's room, and four servants' rooms, together with box rooms, linen room, sanitary offices, etc.

The hospital is to be built so that the whole building can be raised by an additional storey in the future, when it will provide accommodation for something like sixty patients.

With the few points remarked upon, the building is well planned and shows considerable knowledge of the requirements of modern hospitals. The architect is Mr. R. J. Thompson, F.R.I.B.A., of Wimbledon.

## Figures and Tables

**Figure f1:**
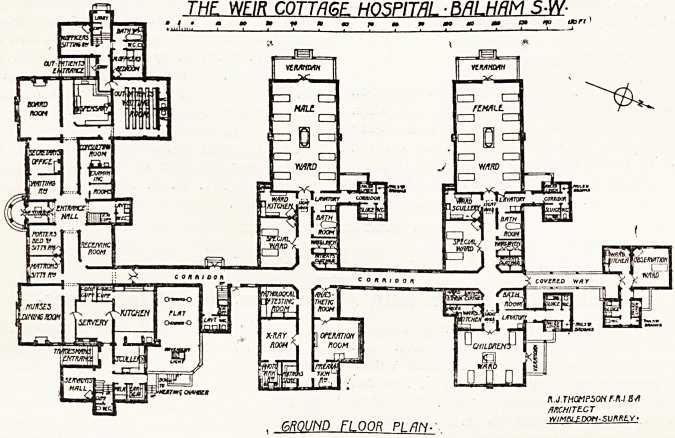


**Figure f2:**